# 289 Trailblazer Pilot Grants as Originators of Research Collaborators: Past, Present, and Future – CORRIGENDUM

**DOI:** 10.1017/cts.2024.541

**Published:** 2024-05-16

**Authors:** Sarah Mejia Glock, Silvia M. Bigatti

The above abstract published with an error in the author list. The third author was shown as Richard M. Fairbanks. This was incorrect and should have been included as part of the affiliation for Indiana University Richard M. Fairbanks School of Public Health.

The original abstract has been corrected online to rectify these errors.
